# Case Report: Severe cutaneous adverse event associated with checkpoint inhibition in the setting of CAR T-cell therapy: beyond CRS

**DOI:** 10.3389/fonc.2023.1171031

**Published:** 2023-06-15

**Authors:** Chiara Masucci, Sara Pepe, Ursula La Rocca, Veronica Zullino, Maria Stefania De Propris, Walter Barberi, Anna Paola Iori, Sabina Martelli, Franco Ruberto, Maurizio Martelli, Alice Di Rocco

**Affiliations:** ^1^ Division of Hematology, Department of Translational and Precision Medicine, Sapienza University, Rome, Italy; ^2^ National Blood Centre, Italian National Institute of Health, Rome, Italy; ^3^ Department of Emergency-Acceptance, Critical Areas and Trauma, Policlinico Umberto 1 Hospital, Rome, Italy; ^4^ Department of General Surgery and Specialist, Sapienza University of Rome, Policlinico Umberto 1 Hospital, Rome, Italy

**Keywords:** CAR T, PMBCL, immunotherapy, CRS, checkpoint inhibitors, bullous erythema

## Abstract

Anti-CD19 chimeric antigen receptor (CAR) T cell therapy actually represents the standard of care for multiple relapsed or refractory primary mediastinal B-cell lymphoma (r/r PMBCL). Checkpoint inhibitors, such as pembrolizumab, appear to be a safe and effective treatment strategy for patients who are ineligible for or resistant to autologous stem cell transplantation. Although preclinical studies suggested that checkpoint inhibitors may enhance the vitality and anti-tumor activity of CAR T cells, there are no substantial/robust clinical data about the immune-mediated toxicity of their association. We describe a case of a severe cutaneous adverse event arising immediately after Cytokine Release Syndrome (CRS) on day +6 from CAR T cells infusion in a young r/r PMBCL patient who previously received pembrolizumab. These skin lesions were interpreted as an immune mediated adverse event, considering their prompt improvement and fully recovering achieved with the addition of immunoglobulin infusion to systemic steroid therapy. This case of life-threatening cutaneous adverse event calls for further investigations about off-target immune-related adverse events deriving from the combination of CAR T cell therapy and checkpoint inhibition, whose synergic therapeutic effect is promising.

## Highlights

- Life-threatening skin adverse event developed after axi-cel infusion in a chemorefractory PMBCL patient following pembrolizumab.
- The serious adverse event of special interest was probably due to the synergic, immune mediated action of CAR T and checkpoint inhibitor.

## Introduction

Primary mediastinal B-cell lymphoma (PMBCL) is an uncommon aggressive subtype of non-Hodgkin lymphoma, with clinical and pathobiological peculiar features (1). Thanks to anthracycline-based chemoimmunotherapy regimens, with or without consolidative radiation, most of newly diagnosed patients can be successfully cured (2–4). In first line therapy refractory patients and in those who relapse after frontline treatment, conventional chemotherapy followed by autologous stem cell transplantation (ASCT) is currently the standard of care (5). However, the outcome of patients with relapsed/refractory PMBCL (r/r PMBCL) is extremely poor (6). The use of checkpoint-inhibitors, as pembrolizumab, seems to be an effective and safe possibility in patients who are ineligible for or refractory to ASCT (7). Moreover, CD19-directed chimeric antigen receptor (CAR) T cell therapy has improved r/r PMBCL prognosis in terms of progression free survival (PFS) and overall survival (OS), since it is approved as third line therapy (8, 9). There are no significant clinical data about the combination of CAR T cells and immune checkpoint blockade, although preclinical studies demonstrated that the addition of programmed death protein 1 (PD-1) inhibitors to CAR T cells therapy promotes vitality and anti-tumor activity of CAR T cells (10). This case report presents the management of a rare adverse event in a young chemorefractory PMBCL patient, treated with pembrolizumab as second-line and then undergoing cellular immunotherapy with CAR T.

## Case presentation

The patient is a 20-year old female with primary refractory stage IIA Bulky PMBCL. PET-CT scan performed at diagnosis showed a highly avid anterior mediastinal mass (SUV 13.3) distorting the superior mediastinum and measuring 110 mm × 100 mm × 85 mm. She was initially treated according to the DA-EPOCH-R immunochemotherapy regimen, receiving 6 cycles. The PET-CT response assessment after 5 weeks from the last cycle revealed no significant metabolic or structural PET response (SUV 10.8, 73 mm × 63 mm, Deauville score 5) consistent with refractory disease. Before second-line therapy, the mediastinal biopsy performed at the time of diagnosis was histopathologically reviewed: PMBCL diagnosis was confirmed, and programmed death-ligand 1 (PD-L1) expression on lymphoma cells was evidenced. Considering her chemorefractory disease and proven PD-L1 overexpression, pembrolizumab was off-label required and obtained. Thus, salvage therapy with pembrolizumab was started at a dose of 200 mg every 21 days. After 4 cycles, the PET-CT scan showed a metabolic reduction of the mediastinal mass (SUV 6), corresponding with a partial response. After the sixth cycle of pembrolizumab, the patient maintained a partial mediastinal response, with no further reduction of PET avidity and a mild increase in metabolic gradient in one of the known active sites (SUV 8), consisting in stable disease. No adverse reaction occurred during the whole treatment period with pembrolizumab.

**Figure 1:**
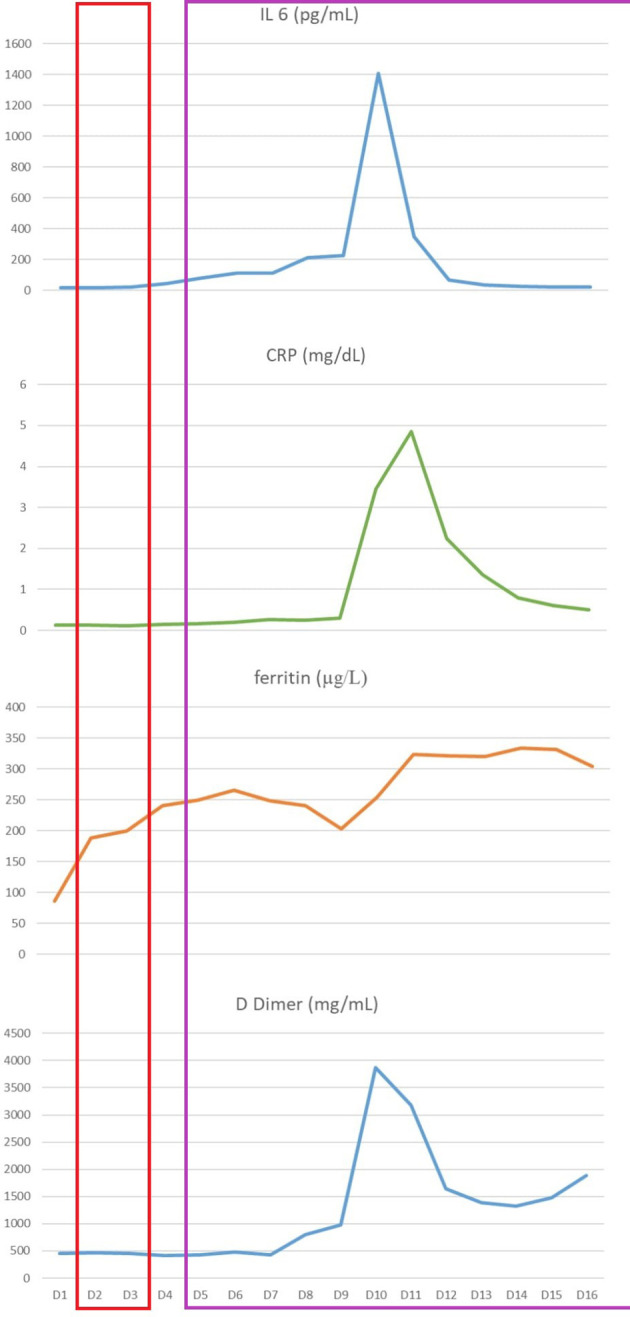
Inflammatory parameters monitoring from day +1 to hospital discharge: interleukin 6 (IL 6), C-reactive protein (CRP), ferritin and D dimer. The red box highlights the CRS period, while the purple one shows the cutaneous adverse event period.

Based on the previous disease course, with an overall chemorefractory biology and a partial metabolic response obtained with immunotherapy, we decided to switch towards an anti-CD19 CAR T cell treatment with axicabtagene ciloleucel (axi-cel). Thirty-four days after the last pembrolizumab administration, lymphocytes were successfully collected. Mediastinal radiation therapy was chosen as bridging therapy at a dose of 30 grays in 10 fractions. Forty-four days after apheresis procedure, the patient received CAR-T therapy. On day 2 patients began to have fever and she started antibiotic therapy and Cytokine Release Syndrome (CRS) monitoring. Fever persisted in the subsequent days, with associated hypotension requiring IV fluids (CRS grade 2). On day 4, she also manifested hypoxia needing oxygen FiO2>40%, so CRS grade 3 was diagnosed and 3 doses of Tocilizumab were administered q8h in combination with dexamethasone 10 mg q6h and she was immediately transferred to the Intensive Care Unit (ICU). CRS signs were followed by a sharp increase in inflammatory parameters as interleukin 6 (IL 6) C-creative protein (CRP), ferritin and D dimer (**Figure 1**). Contextually, a strong CAR T *in vivo* expansion was detected by flow cytometry using an 8-color monoclonal antibody combination to monitor CAR-T cells and lymphocyte subsets (T, B, NK and T-NK cells). CAR T cells were detected by incubation with biotinylated CD19 antigen and FITC-conjugated anti-biotin protein, according to the manufacturers’ instructions (Acro Biosystems, Beijing, China) on peripheral blood samples (from 15.12 CAR T cells/μL on day 3 and 14.7 CAR T cells/μL on day 7 to 36.8 CAR T cells/μL on day 14). The CRS discontinued from day 5 but it was followed by neurotoxicity like immune effector cell-associated neurotoxicity syndrome (ICANS) grade 1, consisting in impaired handwriting. Within the first 8 hours, neurological deficits were spontaneously fully recovered, carrying on steroid therapy.

**Figure 2:**
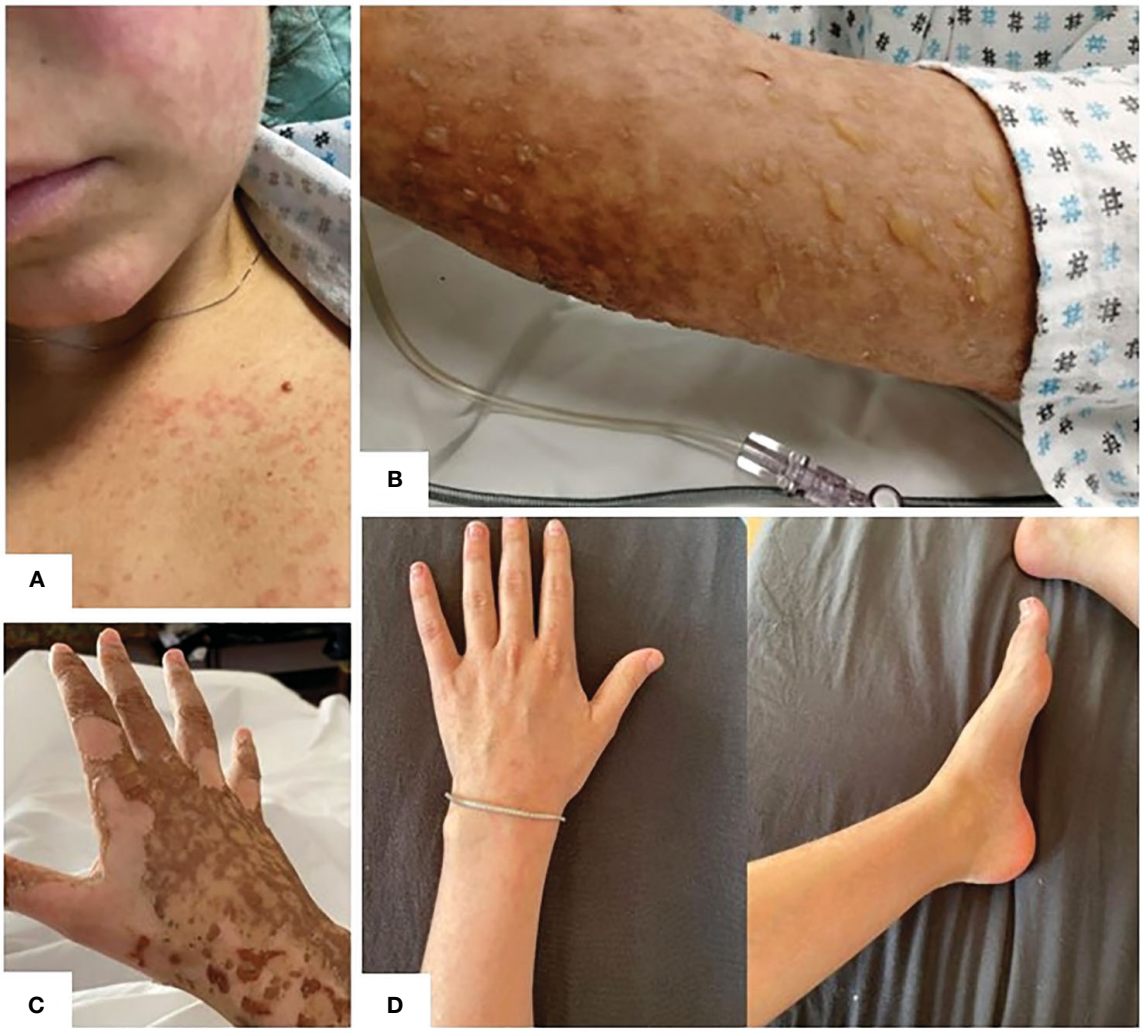
**(A)** Day +6: development of erythematous itchy rash, spreading to malar regions to upper and lower limbs. **(B)** Erythematous-vesicular-bullous lesions affecting upper limbs with brownish hyperpigmented macules on day +8. **(C)** Initial re-epithelization on day +17. **(D)** Complete re-epithelialization 2 months after axi-cel infusion.

On day 6, the patient developed an erythematous itchy rash, spreading to malar regions, chest and upper and lower limbs (**Figure 2A**). Cutaneous lesions rapidly evolved in the subsequent days: skin examination was notable for bullous erythema grade 4 (11) affecting the malar regions and the whole limbs (including palms and soles), with coexisting erosions and brownish hyperpigmented macules (**Figure 2B**), involving about 75 percent of the body surface area. The Nikolsky sign was negative. Firstly, in order to exclude an infectious etiology in an immunosuppressed patient, complete microbiological screening was performed, and antiviral treatment was increased from prophylactic dosage. All the microbiological tests resulted negative. Given the superficial level of desquamation, as well as the onset of the lesions soon after severe CRS and the prolonged course consistent with a more chronic autoimmune presentation, dermatology consultants favored a graft-versus-host disease (GVHD)-like reaction as the primary etiology, discouraging the performing of skin biopsy in consideration of SARS-COV2 pandemic logistics restraints, the intensive care setting and the severe neutropenic state of the patient. Therefore, intravenous high dose immunoglobulin (1 gram/kg daily for 2 consecutive days) were added to the treatment.

Beginning on the day after immunoglobulin infusion, no other skin lesion appeared whereas the remaining began to resolve. (**Figure 2C**). The patient was discharged on day 17 in good clinical condition, with normal complete blood count. Her skin progressively improved and, on medical examination 2 months after axi-cel infusion, re-epithelialization was complete (**Figure 2D**).

**Figure 3:**
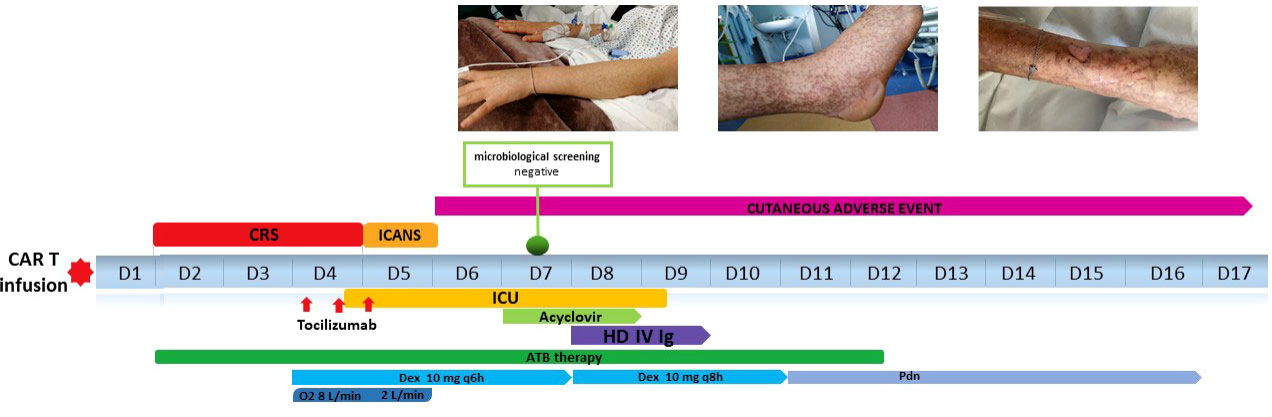
Timeline of relevant clinical data of the patient during hospitalization.

A timeline of relevant clinical data of the patient during hospitalization is showed in **Figure 3**.

After hospital discharge, circulating CAR T cells levels were monitored at day 28 (7.82 cells/μL) and months 3 (2.007 cells/μL) and 6 (28.35 cells/μL).

The PET-CT scans performed at the end of treatment and at 3 and 6 months documented a complete metabolic response. To date, after 22 months from CAR T treatment, the patient remains in complete response and continues follow up every 6 months.

## Discussion

Immune checkpoint inhibitors (ICIs) and CAR T cell therapy are promising methods of immunotherapy, which are becoming increasingly important in r/r PMBCL treatment.

Checkpoint blockade may enhance antitumor immunity and, as a parallel effect, may also increase immune activity against self-antigens, resulting in immune-related adverse events (irAEs) that can affect any body organ (12). These events usually develop within the first few weeks to months after treatment initiation, but they can also present after cessation of ICIs therapy (13). Cutaneous irAEs are the most frequent, often consisting in maculopapular rash and vitiligo, as widely described in oncological settings. Other dermatologic manifestations can also occur, including lichenoid reactions, psoriasis, and autoimmune skin diseases such as bullous pemphigoid and alopecia (14, 15).

Regarding CAR T context, limited and heterogeneous skin manifestations were reported after cellular immunotherapy, such as secondary cutaneous malignancies, cutaneous infections, and “eruption of lymphocyte recovery”, all with no progression features (16).

In our case, skin manifestations broke out in a deep immunosuppression context. Patients treated with CD19 CAR T are at risk of infections due to lymphodepleting therapy, development of CRS, and B cell aplasia with associated hypogammaglobulinemia (17). So, a cutaneous infection secondary to her immunocompromised state was firstly excluded through microbiological screening.

Of particular relevance is the pharmacokinetics of pembrolizumab, which has a long half-life of 26 days (18). In our patient, the leukapheresis product from which the CAR-T cells were made was collected thirty-four days after the last pembrolizumab administration, still in the presence of around 40% of the pembrolizumab dose, and when the cells were infused (79 days from the last dose, about 3 half-lifes) there still would have been 12.5% remaining. Our patient received pembrolizumab initially and then CAR T, with the development of a severe skin manifestation within nine weeks from the last pembrolizumab cycle and six days from CAR T infusion, occurred immediately after severe CRS and ICANS. An immune related cutaneous adverse event was the most plausible hypothesis.

Limited data are actually available about irAEs after combination of immune checkpoint inhibitors and CAR T cells therapy. A similar skin reaction was described in a 22-year-old female with primary refractory PMBCL, treated with pembrolizumab following CAR T therapy, who subsequently developed a maculopapular rash with friable skin and positive Nikolsky sign. Although biopsy was also not performed in this case, it was interpreted as a T cell mediated GVHD-like skin reaction, successfully treated with local and systemic steroids and IV immunoglobulin (19).

GVHD is the immune response of donor T lymphocytes responding to the recipient’s alloantigens.

Some targets of CAR T cells have shared expression on normal tissues and some degree of “on-target/off-tumor” toxicity may occur through engagement of target antigen on nonpathogenic tissues (20). The target antigen of CD-19 directed CAR T cell therapy is restricted to the tumor cell and provides a cytotoxic effect exclusively on the malignant clone. However, the engineered T cells maintain their original T cell receptor and, when activated by exposure to the CD19 antigen, can potentially expand rapidly, damaging normal tissues by recognizing non CD19 antigens that are expressed in those tissues.

In the setting of allogeneic stem cell transplantation, a model of donor T-cell activation by residual anti-PD-1 antibodies in patients previously received ICI was proposed (21). A similar mechanism of amplification of CAR T action on normal tissues antigen may be supposed.

This mechanism may be amplified by the previous administration of checkpoint inhibitors.

No significant clinical data are now available about the synergistic effects of ICIs and CAR T leading to off-target autoimmune effects. Preliminary data from ongoing phase I Zuma-6 trial (NTC02926833) shows that PD-1 inhibition with Atezolizumab after CAR-T cell therapy (axi-cel) has a manageable safety profile, with no significant increased incidence of AEs (22).

To the best of our knowledge, this is the first described case of life-threatening cutaneous adverse event arising immediately after CRS in a lymphoma patient treated with CAR T cells following ICIs therapy. In view of the complete resolution of skin lesions with steroid therapy and IV immunoglobulins, the adverse event described in this case was thought to be immune mediated. Unfortunately, no histological confirmation is available since skin biopsy was not performed.

Our case confirms how the use of immunotherapy is changing the scenario of r/r PMBCL patients, currently considered to have a poor prognosis. However, this report underlines the risk of occurrence of unexpected immune-related adverse events following the sequential use of CAR T and checkpoint inhibitors, requiring prompt identification and immunosuppressive therapeutic management.

## Data availability statement

The original contributions presented in the study are included in the article/supplementary material. Further inquiries can be directed to the corresponding author.

## Ethics statement

Written informed consent was obtained from the individual(s) for the publication of any potentially identifiable images or data included in this article.

## Author contributions

CM and AR participated in the care of the patient, conceived the idea for publication, and wrote the manuscript. SP, UR, VZ, WB, AI, SM and FR participated in the clinical care of the patient and contributed to the manuscript. MP performed the immunophenotypic analysis and contributed to the manuscript. and MM guided the direction of the report, and contributed to the manuscript. All authors contributed to the article and approved the submitted version.
